# Maternal satisfaction among vaginal and cesarean section delivery care services in Bahir Dar city health facilities, Northwest Ethiopia: a facility-based comparative cross-sectional study

**DOI:** 10.1186/s12884-020-03170-w

**Published:** 2020-08-17

**Authors:** Hanna Franco Karoni, Getasew Mulat Bantie, Muluken Azage, Ayele Semachew Kasa, Amare Alamirew Aynie, Gebiyaw Wudie Tsegaye

**Affiliations:** 1 Department of Nursing, Felege Hiwot Referral Hospital, Bahir Dar city, Ethiopia; 2 Department of Public Health, GAMBY Medical and Business College, P.O. Box: +251-209, Bahir Dar city, Ethiopia; 3 School of Public Health, College of Medicine and Health Sciences, Bahir Dar University, Bahir Dar, Ethiopia; 4 Department of Adult Health Nursing, College of Medicine and Health Sciences, Bahir Dar University, Bahir Dar, Ethiopia; 5 Faculty of Community Health, Alkan Health sciences, Business and Technology College, Bahir Dar city, Ethiopia

**Keywords:** Satisfaction, Mothers, Delivery care, Bahir Dar, Ethiopia

## Abstract

**Background:**

Mothers’ delivery care satisfaction is one of the indicators to monitor the quality of health care provision. However, there is only limited information in this regard in Ethiopia, particularly in the study area. Therefore, the study aimed to determine the level of maternal satisfaction and the determinants among vaginal and cesarean section delivery care in Bahir Dar city health facilities.

**Methods:**

Facility-based comparative cross-sectional study was conducted from April to May 2018. Using systematic random sampling, a total of 896 recently delivered mothers were interviewed. The collected data were entered into the Epi-Data soft and then exported to SPSS Version 20.0 for analysis. Descriptive statistics were computed and Logistic regression model was used to identify the association between explanatory and outcome variables. Adjusted Odds Ratio with 95% CI was used to measure the strength of the association between these variables. The model fitness was checked using Hosmer and Lemeshow goodness of fit (*P* > 0.05). A *p*-value < 0.2 at bivariate analysis was considered for variables to be candidates for multivariable logistic regression analysis. Variables with a *p*-value of < 0.05 at multivariate analysis were considered as statistically significant predictors of mothers’ satisfaction.

**Results:**

A total of 894 recently delivered mothers participated in the study yielded a response of 99.8%. 448 (50.1%) mothers delivered vaginally whereas 446 (25.8%) via cesarean section. The overall mean age of respondents was 26.60 (± 4.88) years. The total maternal delivery care service satisfaction level was 61.4%. More mothers were satisfied with vaginal delivery care, 65.6% (95% CI: 56.97, 74.22%) than cesarean section, 57.2% (95% CI: 48.19, 66.2%). Maternal education, residence, current delivery care planned, maternal HIV status, the gender of health care provider and gave birth in a private health facility were significantly associated with vaginal delivery care satisfaction. Whereas, maternal education, residence, current delivery care planned, antenatal care attended, gender of health care provider was significantly associated with cesarean section delivery care satisfaction.

**Conclusions:**

The overall maternal delivery care service satisfaction level was low as, per the national standard, and there is a great discrepancy in maternal satisfaction level between vaginal and cesarean section delivery care services.

## Background

The worldwide maternal mortality ratio (MMR) declined by 44% from 1990 to 2015. However, maternal mortality remains unacceptably high with approximately 303,000 maternal deaths occurring each year, with the largest burden in Sub-Saharan Africa and Asia [1]. Ethiopia is one of the countries with high maternal mortality with an estimated maternal mortality ratio of about 412 per 100,000 live births [2]. Maternal deaths result from a wide range of direct and indirect causes. Accounting 80% of the total maternal deaths, direct causes include hemorrhage, infection, unsafe abortion, hypertensive disorders of pregnancy, and obstructed labor. Many of the maternal deaths could be avoided if preventive measures and cares were taken during the perinatal period [3].

At 2003, Globally, it is estimated that 34% of mothers deliver with no skilled attendant. Skilled attendants’ assistance in developed countries is more than 99% compared to 62% in developing countries [3]. The Ethiopian demographic and health survey (EDHS) 2014 report revealed that the proportion of births delivered with a skilled birth attendant was about 26% [2].

Clients or patients are the ultimate users of a health facility [4]. They expect comfort, care, and cure [5]. After clients come to the hospital, they may become either satisfied or dissatisfied with the service they received [6]. However, their satisfaction is a complex concept that is related to a number of factors, including lifestyle, past experiences, future expectations and on the value of both individual and society [7].

The goal of any service organization is the creation of satisfaction among customers by providing the intended services [8]. Mothers’ satisfaction with delivery care service is a means of secondary prevention of maternal and neonatal mortality during the perinatal period. An increase in the number of mothers who receive a satisfactory delivery care service will increase the subsequent utilization of the service. It also increases the interests of others to receive the service based on a positive recommendation of satisfied clients. Mothers’ satisfaction is also important for mother to infant bonding [9].

The Health Sector Transformation Plan (HSTP) calls for a greater proportion of women to deliver by a skilled attendant [10, 11]. Community-based interventions have been given at grass root level to increase skilled birth attendants through health extension programs since 2003.

However, there is a scanty study of the mode of maternal delivery care satisfactions in Amhara region. Hence, determining the maternal delivery care satisfaction level and identifying its determinants is important to understand the gap and strengthen the existing strategies. Therefore, the purpose of this study was to determine and compare the maternal satisfaction level between vaginal and cesarean section delivery care services and to identify its associated factors in Bahir Dar city health facilities.

## Methods

### Study design, setting, and period

This Facility based comparative cross-sectional study was conducted in Bahir Dar city from April to May 2018. The city is located in Amhara Regional State North West Ethiopia, which is 565 km apart from Addis Ababa, the capital city of Ethiopia. Based on the 2017 population projection, the city had a total population of 321, 343 of which 108, 295 were females. The total number of non-pregnant reproductive age women (15–49 years) was 42,957. There are 9 sub-cities in Bahir Dar city. According to the City Health Department, the health infrastructure of the zone is organized by one governmental specialized referral hospital, one primary hospital, and ten health centers. There are also two private hospitals and ten private clinics. There are four hospitals, one health center and one non-governmental clinic that provides both delivery care services [12].

### Population

All term pregnant mothers who visited dual delivery care health facilities were the source population. And term mothers who visited these health facilities during the data collection period were the study population.

### Sample size determination

The sample size was calculated using the formula for two population proportions with the level of women’s satisfaction between vaginal and cesarean section among the study participants in previous studies was not known; thus, the 10% difference in the proportions between vaginal delivery (P1 = 50%) and cesarean delivery (P2 = 40%), 1:1 ratio between vaginal and cesarean delivery, power = 80%, confidence level = 95% (1.96) was assumed. The formula can be seen below.

$$ {\mathrm{n}}_1=\frac{{\left[{Z}_{\frac{\alpha }{2}}\sqrt{\left(1+\frac{1}{r}\right)P\left(1-P\right)+{Z}_{\beta}\sqrt{P_1\left(1-{P}_1\right)+\frac{P_2\left(1-{P}_2\right)}{r}}}\right]}^2}{{\left({P}_1-{P}_2\right)}^2} $$

By considering a 10% non-response rate, the final sample size was 896.

### Sampling procedure

**Fig. 1:**
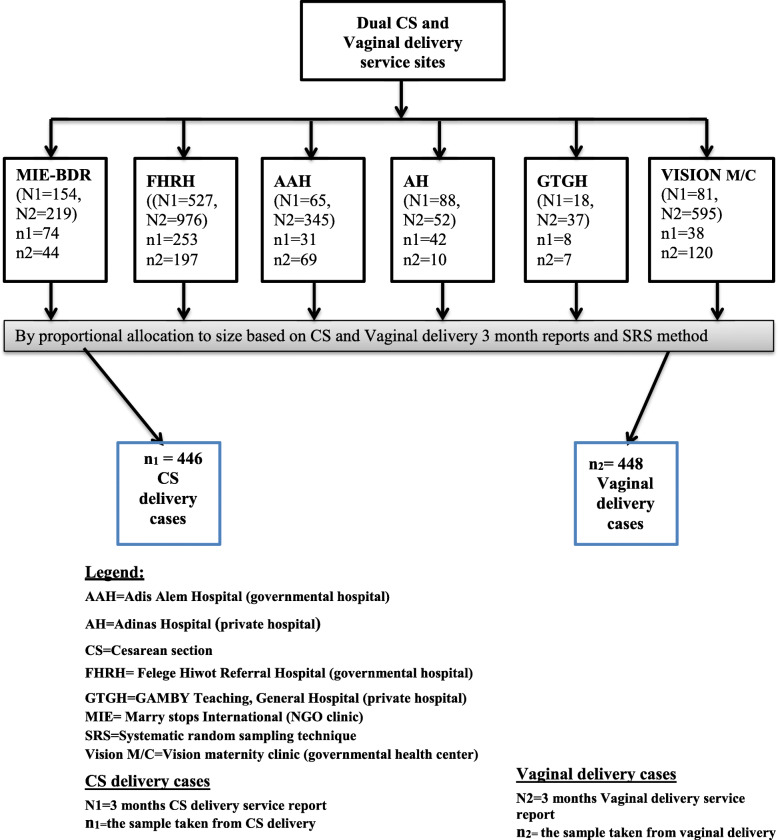
Schematic presentation of the sampling procedure on maternal delivery service satisfaction, Bahir Dar city, 2018

Mothers who delivered in one of the six dual delivery care, providing health facilities were selected for the study. Proportion to size allocation was made to determine the required sample size from each health facility based on the previous 3 months delivery care attendants. The systematic random sampling technique was used until the required sample size was achieved in each health facility. The K^th^ value was calculated based on the number of registered for delivery care on the day divided by expected sample a day (in each health facility). The total number of mothers who gave birth in the study period was 2481, (640 via cesarean section and 1841 via the vaginal route). From the first K^th^ values, one mother was selected by lottery method. The consecutive delivering mother was recruited by every K^th^ value. Finally, from each 896 delivering mothers (448 from the vaginal and 448 from the cesarean section) were selected (Fig. 1).

### Exclusion criteria

Mothers who had a preterm delivery, who lost her consciousness during/after giving birth, and/or who was unable to speak the Amharic language were excluded.

### Variables of the study

#### Dependent variable

Mothers’ satisfaction of delivery care services.

#### Independent variables

##### Socio-demographic characteristics

Age, ethnicity, religion, marital status, educational status, occupation, residence, monthly income.

##### Obstetric related characteristics

Parity, planned current pregnancy, reason to visit the health facility, mode of delivery, fetal birth outcome, and ANC follow up.

##### Maternal delivery service related characteristics

Time spent to get health professionals, presence of waiting area, gender privacy during physical examination, maternal HIV status, mother’s health status after delivery, sex of the baby, the sex of the health professional, greeting during health care provision, respectful practice of professional during delivery, birth weight of the baby, and distance from the health facility.

### Operational definitions

#### Satisfied

We took the Likert scale to measure the satisfaction status of mothers on delivery care services. Each satisfaction assessing question rated from 1 up to 5. Then, we sum up these ten variables altogether. Then, we computed the 75th percentile for the ten variables. Finally, those mothers who scored the value of 75th percentile or more of the satisfaction assessing questions were considered as satisfied of the delivery care services. However, mothers who scored less than the value of 75th percentile were considered as dissatisfied on the delivery care service [9].

#### Vaginal delivery

Vaginal delivery service encompasses spontaneous vaginal, forceps and vacuum delivery.

### Data collection procedure and quality control

Data were collected using a structured questionnaire. The questionnaire was adapted from similar studies. The questionnaire consisted of questions about social-demographic, obstetric, health facility, the health provider and service delivery related questions (attached in the supplementary file portion). Questions related to delivery service satisfaction comprised 10 items with 5 scales Likert type (1- Very Dissatisfied, 2- Dissatisfied, 3-Neutral, 4- Satisfied, 5- Very Satisfied) Before the data collection, the questionnaire was translated into Amharic (national working language) by the independent translator (Ph.D. in linguistics) and then back to English to check for consistency. Finally, the Amharic version was used. The data collectors were two female BSc nurses working in non-selected health facilities and the supervisors were two female health officers working in a private medical college. Then, they took training for 2 days. Pre-test of the questionnaire was done on 85 of delivered mothers in two hospitals having dual delivery care services in the West Gojjam zone. The questionnaire was assessed for clarity, length, and completeness. Then, some adjustment was made in the questionnaire and extra briefing was made to the data collectors.

Then, the actual data were collected over 6 h of giving birth, and a similar questionnaire was administered to both modes of deliveries. The delivery service was assisted by using local (lidocaine) or regional/spinal (mainly bupivacaine) anesthesia.

The daily meeting was held between the principal investigator and the data collectors to detect any problems that had arisen. In addition, inspection for completeness and quality of data collection was carried out daily by supervisors and detailed feedback was provided to data collectors.

### Data processing and analysis

The collected data were checked for completeness and consistency by the principal investigator. Then, they were cleaned, coded and entered into EPI- Data and exported into SPSS Version 20.0 for analysis. Descriptive statistics were computed and Logistic regression model was used to identify the association between explanatory and outcome variables. Adjusted Odds ratio (OR) with 95% CI was used to measure the strength of association between explanatory variables and the outcome variable. The model fitness was checked using Hosmer and Lemeshow goodness of fit (*P* > 0.05). A *p*-value < 0.2 at bivariate analysis was considered for variables to be candidates for multivariable logistic regression analysis. Variables with a *p*-value of < 0.05 at multivariate analysis were considered as statistically significant predictors of mothers’ satisfaction.

### Ethics approval and consent to participate

Ethical approval was obtained from Research and Publication Office of GAMBY Medical and Business College, and the approval letter was obtained from the Amhara public health institute. The college ethics committee approved the procedure for verbal consent as the study is not a sensitive and privacy issue, rather assessing the satisfaction level of delivering mothers in the delivery services they got. The purpose of the study was explained to the respondents and verbal informed consent was obtained from them in the Amharic language. Confidentiality of information was maintained by omitting any personal identifier from the questionnaires. The study participant information sheet was attached to the front page of the questionnaire and before the actual data collection process, the participants were well informed and the data collection was on a voluntary basis. Because we obtained verbal consent, documentation of consent was not required. However, the information provided by each respondent was kept confidential in a secure place.

## Results

### Socio-demographic characteristics of respondents

During the three-month period before data collection, 3157 women were seen in the six dual delivery services with a cesarean rate of 25.8%. 894 recently delivered mothers participated in the study, 448 by vaginal delivery and 446 by cesarean section. The response rate and the overall mean age of the respondents were 99.8% and 26.60 (+ 4.88) years, respectively. 715 (80.0%) respondents were from the Amhara ethnic group and about 603 (68.0%) were Orthodox Christians followers. The majority, 712 (79.6%) were married and about 117 (13.5%) of the respondents were unable to read and write whereas, 321 (35.9%) were graduates. 288 (32.2%) were housewives, while 74 (8.3%) were farmers. 169 (77.2%) mothers came from urban areas and the average monthly household income of delivering mothers was 4096.88 Birr (See Table 1).

**Table 1:** Socio-demographic Characteristics of Delivering Mothers’ in Bahir Dar City, Amhara Regional State, Ethiopia, 2018 (*n* = 894)

Variables	Vaginal delivery		Cesarean section		Total	
	Number	Percent	Number	Percent	Number	Percent
Age (in years)						
< 20 years	27	6.0	34	7.6	61	6.8
20–30	343	76.6	330	74.0	673	75.3
31–49	78	17.4	82	18.4	160	17.9
Ethnicity						
Amhara	362	80.8	353	79.1	715	80.0
Oromo	45	10.0	50	11.2	95	10.6
Tigrie	14	3.1	23	5.2	37	4.1
Agew	27	6.0	20	4.5	47	5.3
Religion						
Orthodox	305	68.1	303	67.9	603	68.0
Muslim	90	20.1	103	23.1	193	21.6
Protestant	50	11.2	38	8.5	88	9.8
Others^a^	3	0.7	2	0.4	5	6.0
Marital Status						
Single	45	10.0	53	11.9	98	11.0
Married	369	82.4	343	76.9	712	79.6
Cohabited	25	5.6	42	9.4	67	7.5
Divorced	4	0.9	3	0.7	7	0.8
Widowed	5	1.1	5	1.1	10	1.1
Educational Status						
Unable to read and write	58	12.9	59	13.2	117	13.5
Able to read and write only	30	6.7	19	4.3	49	5.5
Grade 1–4	18	4.0	9	2.0	27	3.0
Grade5–8	47	10.5	33	7.4	80	8.9
Grade9–10	67	15.0	79	17.7	146	16.3
Grade11–12	71	15.8	83	18.6	154	17.2
Graduates	157	35.0	164	36.8	321	35.9
Occupation						
House wife	124	27.7	139	31.2	288	32.2
Government employee	71	15.8	57	12.8	263	29.4
Farmer	152	33.9	136	30.5	74	8.3
Merchant	29	6.5	45	10.1	128	14.3
Student	18	4.0	21	4.7	39	4.4
Daily laborer	54	12.1	48	10.8	102	11.4
Residence						
Urban	341	76.1	349	78.3	690	77.2
Rural	107	23.9	97	21.7	204	22.8
Monthly Income (in Birr)						
< 1527	122	27.2	101	22.6	223	24.9
1527–3000	122	27.2	117	26.2	239	26.7
3001–5305	111	24.8	98	22.0	209	23.4
> 5305	93	20.8	130	29.1	223	24.9

Other^a^ (catholic, 7th day Adventist)

### Obstetrics characteristics of mothers who delivered in the health facilities

In this study, 380 (42.5%) of mothers gave birth from two to five deliveries. The majority of women 825 (92.3%) had planned pregnancy. The majority of the 831 (93.0%) had at least one ANC visit. However, 17 (1.9%) of the respondents had still birth (See Table 2).

**Table 2:** Obstetrics characteristics of respondents in Bahir Dar city, Amhara Regional State, Ethiopia, April–May 2018 (*n* = 894)

Variables	Vaginal Delivery		Cesarean Section		Total	
	Number	Percent	Number	Percent	Number	Percent
Parity						
One	227	50.7	236	52.9	463	51.8
Two-five	186	41.5	194	43.5	380	42.5
> Five	35	7.8	16	3.6	51	5.7
Reason to visit the health facility						
Planned	248	55.4	248	55.6	496	55.5
Referral	200	44.6	198	44.4	398	44.5
Current pregnancy planned						
Yes	407	90.8	418	93.7	825	92.3
No	41	9.2	28	6.3	69	67.7
ANC Follow Up (at least one visit)						
Yes	410	91.5	421	94.4	831	93.0
No	38	8.5	25	5.6	63	7.0
Fetal Outcome						
Live birth	437	97.5	440	98.7	877	98.1
Still birth	11	2.5	6	1.3	17	1.9

According to the current study, there was a delay to get health professionals for service. About 139 (31.2%) of the cesarean section and 49 (10.9%) of the vaginally delivered mothers spent more than an hour to get the health care worker. Similarly, 382 (85.3%) of vaginally delivered and 313 (70.2%) of the cesarean section delivering mothers had been examined with their privacy kept. 23 (5.1%) of vaginally delivered and 21 (4.7%) of the cesarean section delivering mothers are HIV positive. 361 (80.6%) of vaginally delivered and 296 (66.4%) of the cesarean section delivering mothers faced a health problem after delivery. 97 (21.7%) of vaginally delivered and 45 (10.1%) of the cesarean section delivering mothers gave birth of less than 2500 g baby (See Table 3).

**Table 3:** Health-facility, health care provider, and maternal delivery care service-related characteristics in Bahir Dar city health facilities, Amhara Regional State, Ethiopia, 2018 (*n* = 894)

Variables	Vaginal Delivery		Cesarean section		Total	
	Number	Percent	Number	Percent	Number	Percent
Time spent to get health professionals						
< 1 h	399	89.1	307	68.8	706	79.0
> 1 h	49	10.9	139	31.2	188	21.0
Presence of waiting area						
Yes	325	72.5	270	60.5	595	66.6
No	123	27.5	176	39.5	399	34.4
Gender privacy during physical examination						
Yes	382	85.3	313	70.2	695	77.7
No	66	14.7	133	29.8	199	22.3
Maternal HIV status						
Positive	23	5.1	21	4.7	44	4.9
Negative	364	81.3	375	84.1	739	82.7
Not tested	61	13.6	50	11.2	111	12.4
Mother faced a health problem after delivery						
Yes	361	80.6	296	66.4	657	73.5
No	87	19.4	150	33.6	237	26.5
Sex of the Baby						
Male	238	53.1	258	57.8	496	55.5
Female	210	46.9	188	42.2	398	44.5
The sex of the care provider/professional						
Male	222	49.6	127	28.5	545	61.0
Female	226	50.4	319	71.5	349	39.0
The health worker gave you greeting during health care provision						
Yes	417	93.1	314	70.4	731	81.8
No	31	6.9	132	29.6	163	18.2
The respectful practice of professional during delivery						
Yes	415	92.6	314	70.4	729	81.5
No	33	7.4	132	29.6	165	18.5
Birth weight of the baby (in grams)						
< 2500	97	21.7	45	10.1	142	15.9
2500–4000	345	77.0	393	88.1	738	82.6
> 4000	6	1.3	8	1.8	14	1.6
Distance (in kilometers)						
< 10 km	338	75.4	314	70.4	652	72.9
> 10 km	110	24.6	132	29.6	242	27.1
Walking distance (hours)						
< 2 h	307	68.5	312	70.0	619	69.2
> 2 h	141	31.5	134	30.0	275	30.8

### Maternal satisfaction level in vaginal and cesarean section delivery care services

About half (49.1%) of mothers who delivered vaginally were satisfied with waiting time to see health workers while only 35% of the cesarean section group were satisfied with it. More than one-third of mothers who gave birth via vaginal and cesarean section were very satisfied with maintaining privacy by health staff during care, encouraging and supporting at delivery by health staff, politeness courtesy and respect, availability of medical facilities in the ward, counseling after baby delivered as well as overall cleanness of the facility; while about one fourth of mothers delivered both by vaginal and cesarean section were dissatisfied in the access and cleanness of the toilet, waiting area cleanness and comfort as well as availability of bed in the ward (See Table 4).

**Table 4:** Maternal response on each satisfaction assessing question in delivery care services, Bahir Dar city health facilities, 2018 (*n* = 894)

Variables	Vaginal delivery					Cesarean Section Delivery				
	Very Dissatisfied	Dissatisfied	Neutral	Satisfied	Very Satisfied	Very Dissatisfied	Dissatisfied	Neutral	Satisfied	Very Satisfied
	n (%)	n (%)	n (%)	n (%)	n (%)	n (%)	n (%)	n (%)	n (%)	n (%)
Have you satisfied with waiting time to see health workers	7 (1.6)	45 (10)	13 (2.9)	220 (49.1)	163 (36.4)	18 (4.0)	111 (24.9)	8 (1.8)	154 (34.5)	155 (34.8)
Privacy maintained by health staff during care	6 (1.3)	39 (8.7)	6 (1.3)	224 (50)	173 (38.6)	18 (4.0)	114 (0.9)	4 (25.6)	132 (29.6)	178 (39.9)
Encouraging and supporting at delivery by health staff	9 (2.0)	31 (6.9)	3 (0.7)	225 (50.2)	180 (40.2)	19 (4.3)	105 (23.5)	8 (1.8)	141 (31.6)	173 (38.8)
Politeness courtesy and respect	12 (2.7)	31 (6.9)	5 (1.1)	207 (46.2)	193 (43.1)	19 (4.3)	110 (24.7)	6 (1.3)	132 (29.6)	179 (40.1)
Availability of medical facility in the ward	19 (2.7)	55 (6.9)	20 (1.1)	176 (46.2)	178 (43.1)	20 (4.5)	71 (15.9)	13 (2.9)	166 (37.2)	176 (39.5)
Advice and counseling after your baby delivered	16 (3.6)	53 (11.8)	11 (2.5)	195 (43.5)	173 (38.6)	20 (4.5)	107 (24)	11 (2.5)	145 (32.5)	163 (36.5)
Overall cleanliness of the facility	28 (6.2)	82 (18.3)	15 (3.3)	166 (37.1)	157 (35)	29 (6.5)	137 (30.7)	12 (2.7)	110 (24.7)	158 (35.4)
Access and cleanness of the toilet	45 (10)	113 (25.2)	7 (1.6)	142 (31.7)	141 (31.5)	72 (16.1)	148 (33.2)	3 (0.7)	79 (17.7)	144 (32.3)
Waiting area cleanness and comfort	33 (7.4)	105 (23.4)	10 (2.2)	148 (33)	152 (33.9)	34 (7.6)	143 (32.1)	8 (1.8)	100 (22.4)	161 (36.1)
Availability of bed in the ward	44 (9.8)	111 (24.8)	2 (0.4)	134 (29.9)	157 (35)	40 (9.0)	146 (32.7)	6 (1.3)	92 (20.6)	162 (36.3)

**Fig. 2:**
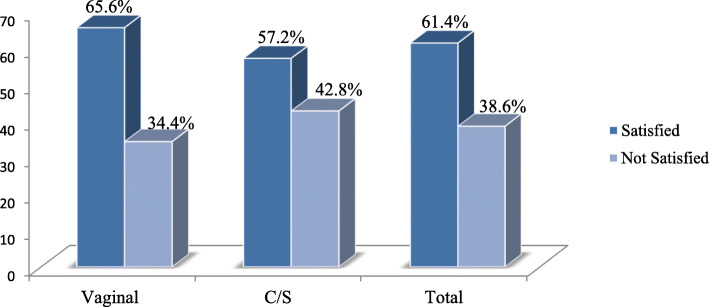
Maternal satisfaction level with respect to the mode of delivery care services at Bahir Dar city health facilities, North West Ethiopia, 2018 (*n* = 894)

By taking ten satisfaction assessing questions and determining the composite index value, the total satisfaction level of mothers who delivered at Bahir Dar city health facilities was 61.4%. The majority of mothers was satisfied more through vaginal delivery, 65.6% (95% CI: 56.97, 74.22%). While only 57.2% (95% CI: 48.19, 66.2%) of mothers who delivered through cesarean section were satisfied (See Figs. 1 and 2).

### Factors associated with maternal satisfaction at vaginal delivery care services

Multivariable logistic regression analysis was conducted to identify independent predictors of maternal satisfaction with a vaginal delivery care service. On multivariable logistic regression model educational status, residence, reason to visit health institution, maternal HIV status, antenatal care follows up, and gender of health care workers, and health facility ownership associated with maternal satisfaction at *p*-value of 0.05.

Accordingly, those mothers who are unable to read and write and 1–4 graded, the odds ratio of satisfaction with vaginal delivery service was about 2.42 (AOR = 2. 42, 95% CI: 1.14, 5.14) and 5.52 (AOR = 5. 52, 95% CI: 1.14, 26.74) compared to those who had a diploma and above. Mothers who were from urban residence, the odds ratio of vaginal section delivery service satisfaction was 2.1 times higher (AOR = 2.01, 95% CI: 1.21, 3.54) compared to those who was from rural residents. Those who visit health institutions for the current delivery care was planned, the odds ratio of cesarean section delivery service satisfaction was 2.1 times higher (AOR = 2.08, 95% CI: 1.28, 3.39) compared to those who referred. Mothers whose HIV status were not tested, the odd of delivery care satisfaction was 0.23 times lower (AOR = 0. 23; 95% CI:0.07, 0.73) compared to those who were tested.

Those mothers who got delivery care services by female physician, the odds of cesarean section delivery service satisfaction were 2 times higher (AOR = 1.99, 95% CI: 1.25, 3.18) than mothers who got the service by male physicians. Those mothers who gave birth in private health facilities, the odds of delivery care satisfaction were about 6 times higher (AOR = 6. 01; 95% CI: 2.01, 17.96) compared to governmental health facilities (See Table 5).

**Table 5:** Factors associated with maternal satisfaction with vaginal delivery care services (VDCS) in Bahir Dar city health facilities, 2018 (*n* = 448)

Variables	VDCS satisfaction status		COR at 95% CI	AOR at 95% CI
	Satisfied	Dissatisfied		
Educational status				
Unable to read and write	38	20	0.91 (0.48, 1.73)	**2.42 (1.14, 5.14)** *
Able to read and write only	21	9	1.12 (0.48, 2.62)	2.52 (0.97, 6.54)
Grade 1–4	16	2	3.85 (0.85, 17.38)	**5.52 (1.14, 26.74)**
Grade 5–8	35	12	1.40 (0.67, 2.93)	1.91 (0.85, 4.29)
Grade 9–10	41	26	0.76 (0.42, 1.37)	1.28 (0.65, 2.52)
Grade 11–12	37	34	**0.52 (0.29, 0.93)**	0.84 (0.43, 1.62)
Graduates ^R^	106	51	1.00	1.00
Residence				
Urban	241	100	**2.45 (1.57, 3.83)**	2.01 (1.21, 3.54)**
Rural ^R^	53	54	1.00	1.00
Reason to visit health institutions for the current delivery care				
Planned	192	56	**3.29 (2.19, 4.95)**	2.08 (1.28, 3.39)*
Referral ^R^	102	98	1.00	1.00
Number of children				
1 child	139	88	**0.26 (0.09,0.70)**	0.50 (0.17, 1.47)
2–5 child	125	61	**0.34 (0.13,0.92)**	0.630 (0.21, 1.85)
> 5 child ^R^	30	5	1.00	1.00
Planned current pregnancy				
Yes	278	129	**3.37 (1.74, 6.52)**	1.85 (0.86, 3.99)
No ^R^	16	25	1.00	1.00
ANC follows up				
Yes	280	130	**3.69 (1.85, 7.37)**	0.95 (0.35, 2.60)
No ^R^	14	24	1.00	1.00
Fetal Outcome				
Live birth	288	149	1.61 (0.48, 5.36)	
Still birth ^R^	6	5	1.00	1.00
Maternal HIV status				
Positive ^R^	16	7	1.00	1.00
Negative	258	106	1.07 (0.43, 2.66)	0.84 (0.30, 2.29)
Not tested	20	41	**0.21 (0.08, 0.60)**	0.23 (0.07, 0.73)*
The sex of the health care provider				
Female	169	53	**2.58 (1.72, 3.86)**	1.99 (1.25, 3.18) *
Male ^R^	125	101	1.00	1.00
Health facility ownership				
Private	53	4	**8.25 (2.93, 23.25)**	6.01 (2.01, 17.96) **
Governmental ^R^	241	150	1.00	1.00

Note: * statistically significant at *P* < 0.05, ** < 0. 01, *** < 0.001; ^R^reference category

### Factors associated with maternal satisfaction with cesarean section delivery care services

Simple logistic regression analysis was conducted to identify independent predictors of maternal satisfaction with cesarean section delivery care services. On multivariable logistic regression model educational status, residence, reason to visit health institution, antenatal care follows up and gender of health care workers associated with maternal satisfaction at *p*-value of 0.05.

Accordingly, those mothers who are solely able to read and write, the odds ratio of satisfaction with cesarean section delivery service was about 10.3 (AOR = 10. 3, 95% CI: 2.15, 49.50) times higher compared to graduates. In contrast, mothers who attended 9 up to 10 and 11 up to 12 grades, the odds ratio of cesarean section delivery service satisfaction was 0.5 lower (AOR = 0. 5, 95% CI: 0.25, 0.97) than graduates.

Mothers who were from urban residence, the odds ratio of cesarean section delivery service satisfaction was 3.6 times higher (AOR = 3.65, 95% CI: 1.84, 7.25) compared to those who was from rural residents. Those who visit health institutions for the current delivery care was planned, the odds ratio of cesarean section delivery service satisfaction was 7.8 times higher (AOR = 7.76, 95% CI: 4.69, 12.84) compared to those who referred. Those mothers who attended antenatal care, the odds ratio of cesarean section delivery service satisfaction was 5 times higher (AOR = 5. 03, 95% CI: 1.35,18.75) than mothers who did not attend antenatal care.

The sex of the health care provider strongly affected the level of mothers’ satisfaction during delivery care services. Those mothers who got delivery care services by female physician, the odds of cesarean section delivery service satisfaction were 1.8 times higher (AOR = 1.79, 95% CI: 1.03, 3.12) than mothers who got the service by male physicians (See Table 6).

**Table 6:** Factors associated with maternal satisfaction with Cesarean section delivery care services (CSDCS) in Bahir Dar city health facilities, 2018 (*n* = 446)

Variables	CSDCS satisfaction status		COR at 95% CI	AOR at 95% CI
	Satisfied	Dissatisfied		
Educational status				
Unable to read and write	29	30	**0.45 (0.25,0.82)**	1.57 (0.68, 3.61)
Able to read and write only	16	3	2.48 (0.69, 8.87)	10.32 (2.15, 49.50)**
Grade 1–4	5	4	0.58 (0.15, 2.25)	1.61 (0.25, 10.28)
Grade 5–8	19	14	0.63 (0.29, 1.35)	1.84 (0.69, 4.89)
Grade 9–10	34	45	**0.35 (0.20,0.61)**	0.49 (0.25, 0.97)*
Grade 11–12	40	43	**0.43 (0.25,0.74)**	0.51 (0.26, 0.97)*
Graduates ^R^	112	52	1.00	1.00
Occupational status				
Government employee	81	58	0.84 (0.43, 1.65)	0.54 (0.21, 1.36)
Merchant	39	18	1.30 (0.58, 2.92)	0.59 (0.21, 1.71)
Housewife	90	46	1.17 (0.59, 2.33)	0.91 (0.36, 2.25)
Farmer	9	36	**0.15 (0.06,0.38)**	0.71 (0.17, 2.91)
Student	6	15	**0.24 (0.08, 0.73)**	0.24 (0.06, 1.11)
Daily laborer ^R^	30	18	1.00	1.00
Monthly Income (in Birr)				
< 1527	55	46	0.59 (0.35, 1.01)	1.07 (0.51, 2.28)
1527–3000	53	64	**0.41 (0.24,0.69)**	0.53 (0.27, 1.04)
3001–5305	60	38	0.78 (0.45, 1.35)	0.65 (0.33, 1.29)
> 5305 ^R^	87	43	1.00	1.00
Residence				
Urban	230	119	**5.56 (3.36, 9.23)**	3.65 (1.84, 7.25)***
Rural ^R^	25	72	1.00	1.00
Reason to visit health institutions for the current delivery care				
Planned	199	49	**10.3 (6.63, 15.98)**	7.76 (4.69, 12.84)*
Referral ^R^	56	142	1.00	1.00
The current pregnancy, planned				
Yes	247	171	**3.61 (1.55, 8.39)**	0.51 (0.10, 2.55)
No ^R^	8	20	1.00	1.00
ANC follows up				
Yes	252	169	**10.93 (3.22, 37.11)**	5.03 (1.35, 18.75)*
No ^R^	3	22	1.00	1.00
Fetal Outcome				
Live birth	252	188	1.34 (0.27, 6.71)	
Still birth ^R^	3	3	1.00	1.00
Sex of the health care provider				
Female	91	36	**2.39 (1.53, 3.72)**	1.79 (1.03, 3.12)*
Male ^R^	164	155	1.00	1.00

Note: * statistically significant at *P* < 0.05, ** < 0.01, *** < 0.001; ^R^ reference category

## Discussion

This study determined the level of maternal satisfaction between vaginal and cesarean section delivery care services and identified the associated factors at the health institutions. The study revealed that 65.6% of mothers who delivered vaginally and 57.2% who delivered through cesarean section were satisfied with delivery care services. This finding was supported by a study in Hawassa city, South Ethiopia [13]. However, the current study contradicted from a study done in Debre Markos, Ethiopia [14].

The study revealed that about one-fourth of delivering mothers was disagreed on the access and cleanness of the toilet, waiting area cleanness and comfort as well as the availability of a bed in the ward. This finding was consistent with the study finding of Nepal [15]. The possible justification for this poor and inconvenient of delivering care services might be due to immature health care managers and /or lack of enough financial expenditure to maintain the quality service of maternal health.

The overall maternal satisfaction level (61.4%), this study finding was in line with a study conducted in Amhara Region referral hospitals, Ethiopia, 61.9% [9]. However, it was higher than studies conducted in Sirilanka 30.4% [16], Nairobi, Kenya, 56% [17], and Addis Ababa Ethiopia, 19% [18]. The possible reason for this discrepancy might be women’s perception towards satisfaction, the management approach of the health managers of the health institutions and the staffing of the health institutions. In contrast, the current study finding was lower than a study done in Debre Markos town, Northwest Ethiopia, 81.7% [14]. The current study held in the city, at which the city residents, might have better access to health information, communication which might enforce them to expect more services beyond what they got. As a result, it might inversely affect their satisfaction.

In addition to determining the magnitude of maternal delivery care service satisfaction, the current study also identified various maternal delivery care service satisfaction, predictors. Mothers who were not able to read and write and 1–4 graded were more likely to be satisfied with vaginal delivery care. Similarly, mothers who solely read and write and gave birth via C/S were more satisfied as compared to those who had a diploma and above. In contrast, mothers who were 9–12 graded were less likely to be satisfied. This result is supported by studies done in Hawassa city, South Ethiopia [13] and in Italy [19]. This could be explained by the tendency that more educated women might have a better understanding of health information, communication and might lead them to expect more services in the health institution which in fact diminishes the perception of maternal satisfaction.

Our study has revealed that an ANC followed up was associated with five times increased odds of satisfaction with cesarean section delivery care compared to mothers who didn’t follow up. This could be mothers were more advised on the importance of operational delivery during the follow up period. Moreover, mothers who were from urban area were more satisfied both in the vaginal and C/S delivery care services as compared to those who were from rural areas. The possible justification for this might be, the urban mothers are near to the health information, communication and they are open for discussion with the physicians and do not afraid / ashamed of privacy or procedure related issues as they waiting for their baby regardless of the route of delivery. In contrast, the rural mothers are mostly not educated and they are ashamed of their privacy and procedural related issues as they prefer to deliver at their home.

Maternal HIV status was one of the exploratory variables for vaginal delivery care satisfaction, but not in the case mothers who delivered through cesarean section. Mothers who were not tested for HIV during delivery were negatively correlated with vaginal delivery care service satisfaction. This may be mothers whose sero-status were unknown were frustrated the intrapartal HIV transmission which might affect the owned satisfaction perception towards delivery care services.

The other significant predictor of maternal delivery care service satisfaction was the gender of delivery care provided health worker. The study revealed that mothers who got services by female health care workers were more satisfied with vaginal and C/S delivery care. This result is supported by studies done in Hawassa city, southern Ethiopia [13]. This may be because female delivery care attendants may assist delivering mothers’ as per the mother’s interest and may respect her cultural norms and engage in activities like the coffee ceremony and porridge preparation while mother laboring; this might give her great opportunity to be more satisfied.

Health facility ownership had also shown a significant association with vaginal delivery care service satisfaction. The likelihood of mothers who delivered vaginally in private health institutions was more likely satisfied than in governmental health institutions. This might be due to the fact that in the Ethiopian context private health institutions are more equipped with skillful health experts and very standard medical instruments which might lead them to have more satisfaction. In addition, health care workers in private health institutions might give close follow up and psychological support for laboring mothers which may make mothers more satisfied. However, mothers who delivered by cesarean section in private health institutions was not satisfied. This might be the private health institutions request much more money than the governmental health institutions. Which in fact might make the mothers, not satisfied delivering by cesarean section in private health institutions.

Reason to visit health institutions for delivery care services were also the other determinant factor for maternal satisfaction with vaginal and cesarean section delivery care services. Mothers who visited health facilities for delivery care services in a planned manner were more likely to be satisfied as compared to mothers who came to health facilities through referral linkage from lower healthcare units. This is consistent with a study conducted in Debre Markos, Ethiopia, [14]. The possible justification for this might be that those mothers who planned to give birth in the health institutions were more informed about the delivery modalities and their pros and cons which might increase their confidence in C/S delivery care service which in turn enhances maternal satisfaction.

### Strength and limitation of the study

Even though this study has provided valuable evidence regarding the level of maternal delivery care service satisfaction and possible associated factors, it could not avoid the chicken-egg dilemma. There was also a challenge of having standard measuring cut-point for maternal delivery care satisfaction. Similarly, the interview held after the cesarean section operation was performed. But did not consider the satisfaction of mothers during the antepartum cesarean section, which was also the other limitation of this study.

## Conclusion

The overall maternal delivery care service satisfaction level was found to be low as, per the national standard, and there was also a great discrepancy in maternal satisfaction level between vaginal and cesarean section delivery care services. Maternal education, residence, current delivery care planned, maternal HIV status, the gender of health care provider and gave birth in a private health facility had a significant association with vaginal delivery care satisfaction. Whereas, maternal education, residence, current delivery care planned, antenatal care attended, the sex of the health care provider were significantly associated with maternal satisfaction with cesarean section delivery care services.

## Supplementary information

**Additional file 1.** English version questionnaire.

## Data Availability

The data can be accessed from the corresponding author upon justified request.

## Abbreviations

ANC: Antenatal Care
AOR: Adjusted Odds Ratio
C/S: Cesarean Section
CI: Confidence Interval
EPI-DATA: Epidemiological Data
EDHS: Ethiopian Demographic and Health Survey
MMR: Maternal Mortality Rate
OR: Odds Ratio
PNC: Postnatal Care
WHO: World Health Organization
SPSS: Statistical Package for Social Science Study
SVD: Spontaneous Vaginal Delivery
