# Supplementary material for: Maternal satisfaction among vaginal and cesarean section delivery care services in Bahir Dar city health facilities, Northwest Ethiopia: a facility-based comparative cross-sectional study
Source: BMC Pregnancy Childbirth. 2020 Aug 17;20:473. doi: 10.1186/s12884-020-03170-w (PMC7433055; doi:10.1186/s12884-020-03170-w)
Supplement: Supplementary file 1 — Additional file 1. English version questionnaire. [file 12884_2020_3170_MOESM1_ESM.docx]

**Supplementary file**

**English version questionnaire**

**Part One: Socio-Demographic Characteristics**

| No. | Question | Category/Answer |
| --- | --- | --- |
| 101 | Age of the respondent (in years) | …..….. |
| 102 | What is your ethnicity? | 1. Amhara 2. Oromo 3. Tigrie 4. Other (if any, specify) ____ |
| 103 | What is your Religion? | A. Orthodox  B. Muslim  C. Protestant  D. Others (if any, specify)____ |
| 104 | What is your Marital status? | A. Single  B. Married  C. Cohabited  D Divorced  E. Widowed |
| 105 | What is your educational level? | A. able to read and write  B. unable to read and write  C. Grade 1-4  D. Grade 5-8  E. Grade 8-10  F. Grade 11-12  G. diploma and above |
| 106 | What is your occupation? | 1. House wife 2. Government employee 3. Farmer 4. Merchant 5. Student 6. Daily laborer |
| 107 | Where is your residence? | 1. Urban 2. Rural |
| 108 | Monthly Income (in Birr) | _______ |
| 109 | For how long did you wait to see a doctor or Nurse? | A. < 1hour  B. >1hour |
| 110 | Did you perceive presence of waiting area? | A. Yes  B. No |
| 111 | Was your privacy kept during examination? | A. Yes  B. No |

**Part-Two-Obstetric-Related Characteristics**

| No. | Question | Category/Answer |
| --- | --- | --- |
| 201 | How many children did you give birth including the current one? | …..….. Births |
| 202 | What was the reason to visit this Hospital? | A. Planned delivery  B. Referral delivery |
| 203 | Was the current pregnancy Planned? | A. Yes  B. No |
| 204 | Did you have ANC follow-up (at least one visit)? | A. Yes  B. No |
| 205 | What was the mode of delivery? | A. Spontaneous vaginal delivery  B. Assisted delivery  C. Cesarean delivery |
| 206 | What was the fetal outcome? | A. Alive  B. Stillbirth |
| 207 | What was the sex of the baby? | A. Male  B. Female |
| 208 | What was your HIV status? | A. positive  B. negative |

Part-Three-Health-facility, health care provider, and maternal delivery care service-related characteristics

| No. | Question | Category/Answer |
| --- | --- | --- |
| 301 | Time spent to get health professionals (in hours) | ___________ |
| 302 | Presence of waiting area | 1. Yes 2. No |
| 303 | Gender privacy during physical examination | 1. Yes 2. No |
| 304 | Maternal HIV status | 1. Positive 2. Negative 3. Not tested |
| 305 | Mother faced a health problem after delivery | 1. Yes 2. No |
| 306 | Sex of the Baby | 1. Male 2. Female |
| 307 | The sex of the care provider/professional | 1. Male 2. Female |
| 308 | The health worker gave you greeting during health care provision | 1. Yes 2. No |
| 309 | The respectful practice of professional during delivery | 1. Yes 2. No |
| 310 | Birth weight of the baby (in grams) | _______________ |
| 311 | Distance to arrive to the health institution (in kilometer) | _______________ |
| 312 | Time taken to arrive the health institution (in hour) | _______________ |

**Part Four- Maternal Satisfaction Related Questions**

Encircle the appropriate response in the following manner for the following questions.

1=Very dissatisfied; 2=Dissatisfied;3=Neutral;4=Satisfied; 5=Very satisfied

| No. | Question | Category/Answer |
| --- | --- | --- |
| 401 | Waiting time to see Health worker. | 1. Very dissatisfied 2. Dissatisfied 3. Neutral 4. Satisfied 5. Very satisfied |
| 402 | Privacy maintained by Health staff during care. | 1. Very dissatisfied 2. Dissatisfied 3. Neutral 4. Satisfied 5. Very satisfied |
| 403 | Encouragement and support at delivery by Health staff. | 1. Very dissatisfied 2. Dissatisfied 3. Neutral 4. Satisfied 5. Very satisfied |
| 404 | Politeness, Courtesy and respect shown by the health care provider. | 1. Very dissatisfied 2. Dissatisfied 3. Neutral 4. Satisfied 5. Very satisfied |
| 405 | Availability of Medical facility in the ward (Drugs, Equipment). | 1. Very dissatisfied 2. Dissatisfied 3. Neutral 4. Satisfied 5. Very satisfied |
| 406 | Health advice and information given by the staff to look after your baby. | 1. Very dissatisfied 2. Dissatisfied 3. Neutral 4. Satisfied 5. Very satisfied |
| 407 | Over all cleanness of the facility | 1. Very dissatisfied 2. Dissatisfied 3. Neutral 4. Satisfied 5. Very satisfied |
| 408 | Access and cleanness of toilet. | 1. Very dissatisfied 2. Dissatisfied 3. Neutral 4. Satisfied 5. Very satisfied |
| 409 | Waiting area cleanness and comfort. | 1. Very dissatisfied 2. Dissatisfied 3. Neutral 4. Satisfied 5. Very satisfied |
| 410 | Availability of beds in the ward. | 1. Very dissatisfied 2. Dissatisfied 3. Neutral 4. Satisfied 5. Very satisfied |

Interviewer Name……………………………………date……………

Supervisor Name…………………………………… date……………

**Thank you very much for your time and participation!!**
